# Ethnicity and incidence of Hodgkin lymphoma in Canadian population

**DOI:** 10.1186/1471-2407-9-141

**Published:** 2009-05-11

**Authors:** Punam Pahwa, Chandima P Karunanayake, John J Spinelli, James A Dosman, Helen H McDuffie

**Affiliations:** 1 Canadian Centre for Health and Safety in Agriculture, University of Saskatchewan, Royal University Hospital, 103 Hospital Drive, Saskatoon, SK, S7N 0W8, Canada; 2 Cancer Control Research, British Columbia Cancer Agency, 2-111, 675 West 10th Avenue Vancouver, British Columbia, V5Z 1L3, Canada; 3 Department of Community Health & Epidemiology, University of Saskatchewan, Health Science Building, 107, Wiggins Road, University of Saskatchewan, Saskatoon, Saskatchewan, S7N 5E5, Canada

## Abstract

**Background:**

Research has shown that ethnicity is a significant predictor of Hodgkin lymphoma (HL). Variations in cancer incidence among ethnic groups in the same country can lead to important information in the search for etiological factors. Other risk factors important in the etiology of HL are medical history and exposure to pesticides. In this report we investigated the association between ethnicity and HL in the presence of medical history, and exposure to pesticides.

**Methods:**

The data resulting from a matched population-based case-control study conducted in six provinces of Canada (Ontario, Quebec, Manitoba, Saskatchewan, Alberta, and British Columbia) was analyzed to determine whether or not there was any association between ethnicity and incidence of HL when adjusted for personal medical history and pesticide exposure. Information on ethnicity, personal medical history, and pesticide exposure was collected by questionnaires via mail on 316 men diagnosed with HL; and on 1506 controls. A conditional logistic regression was utilized and results were presented as odds ratios and 95% confidence intervals.

**Results:**

In our study population, the distribution of ethnic groups was: 38.5% North American, 15% British, 8.4% Western European, 8.2% Eastern European, 1.7% Asian, 1.4% Scandinavian and 27% of other ethnic origin. Compared to North Americans (i) the risk of HL was greater among the Eastern European descendents (Odds Ratio (OR_adj_): 1.82; 95% confidence interval (CI): 1.02, 3.25) and Western European (OR_adj_: 1.62; 95% CI: 0.95–2.76) descent population (borderline significance at 5% level); and (ii) the risk of HL was lower in Asian descents. Diagnosis with measles (OR_adj_: 0.72, 95% C.I.: 0.53–0.98) and/or positive history of allergy desensitization shots (OR_adj_: 0.55, 95% C.I.: 0.30–0.99) were negatively associated with the incidence of HL, while diagnosis with acne (OR_adj_: 2.12, 95% C.I.: 1.19–3.78), shingles (OR_adj_: 2.41, 95% C.I.: 1.38–4.22) and positive family history of cancer (OR_adj_: 1.93, 95% C.I.: 1.40–2.65) increased the risk of HL. Exposure to individual herbicide dichlorprop showed an increased risk of HL (OR_adj_: 6.35, 95% C.I.: 1.56–25.92).

**Conclusion:**

In Canada, compared to North Americans descendents, the risk of HL was significantly greater among the Eastern European and Western European descent population. Our results related to association between ethnicity and HL support the findings reported by other researchers. Our data showed that subjects who were diagnosed with measles or had allergy desensitization shots negatively associated with the incidence of HL; and other medical conditions, ever diagnosed with acne, and positive family history of cancer were positively associated with the incidence of HL.

## Backround

Hodgkin lymphoma (HL) is a specialized form of lymphoma. HL has characteristics that distinguish it from all other cancers of the lymphatic system. An abnormal cell called the Reed-Sternberg cell, a large and malignant cell is found in the HL tissues [1]. HL is a complex of related conditions that are, in part mediated by genetic susceptibilities, infectious diseases, and immune deficits [1]. HL has been increasing in incidence in many countries around the world, particularly in the West [2]. Incidence rates have been increasing in adolescents and young adults more than in any other age group. Recently it is reported that all HL subtypes pooled, has a bimodal peak at ages 15–34 years and over 60 years in most European, American, Hispanic and Australian populations, and Jews born in Israel with incidence rates of around 6 per 100 000 per year in the over-60-year-olds. However, cases with Asian origin show few cases at all in the younger ages; rates increase with age but the highest rates are only half that of the European disease [1]. The finding of association between ethnicity and increased risk of HL has been reported by other researchers as well [3,4]. Ethnicity in health research helps us to understand causes of disease and, particularly the relative contributions of genetic and environmental factors [5-10]. Variations in cancer incidence and ethnic groups in the same country can lead to some important information about the search of etiological factors. The distribution of ethnic groups varies significantly among different regions of Canada.

A study conducted in the United States compared the occurrence of HL, in Chinese, Japanese, Filipino and Asian Indian populations living in the United States to the occurrence in similar groups living in Asia and observed that incidence rates were quite low in all Asian subgroups, but approximately double in United States Asians compared to in native Asians [11]. Stiller et al. [12] suggest that the occurrence of HL in early childhood is related more to ethnicity than to geographical location and may reflect genetic factors or environmental exposures specific to the lifestyle of particular ethnic groups.

Recently, McDuffie et al. [13] and Hoppe et al [14] reported that there are several putative risk factors for HL, which include occupational exposure to certain chemicals and specific pesticides, personal history of certain cancers and other medical conditions, such as infectious disease (bacterial and viral), immune related disorders including autoimmune (e.g. rheumatoid arthritis) and chronic inflammatory diseases (e.g. inflammatory bowel disease), and tonsillectomy. Some antecedent selected medical conditions have been positively associated with HL and other cancers [15-18], while there are some inconsistent findings. Several studies have shown an inverse relationship between prior allergies and cancer [19-23].

Zahm et al [24] reported that there is some genetic resistance to HL in Asians but environmental factors, such as agricultural exposures have some influence as well. Exposure to pesticides has been associated with an increased incidence of Hodgkin lymphoma in many studies, but not in all. A Swedish study found a non significant increased risk of HL in those who have been exposed to phenoxy acid herbicides [25,26]. Another Swedish study showed that exposure to phenoxy acid herbicides has been associated with increased risk of HL [27]. Hardel et al. [28] reported that combined exposure to phenoxy acids and chlorophenols increase the risk of HL. A study in Kansas [24] found no association among men between HL and use of insecticides on crops or on animals. Swedish study of pesticides applicators did not found any increased risk of HL [29]. Another cohort study conducted among workers at a phenoxyherbicide-manufacturing plant in Germany did not find significantly high risk of HL [30].

In this report, we investigated the association between ethnicity and incidence of HL in the presence of medical history, and exposure to pesticides.

## Methods

Details of the study design and methodology have been previously published [31,32]. Briefly, we conducted a matched population based case-control study of men resident in six Canadian provinces (Quebec, Ontario, Manitoba, Saskatchewan, Alberta, and British Columbia) to test the pesticide-exposure hypothesis related to four rare tumors (Hodgkin's Lymphoma (HL), Non-Hodgkin's Lymphoma (NHL), Multiple Myeloma (MM), and Soft-Tissue sarcoma (STS)). There were total 1528 cases diagnosed with four types of tumours under investigation (HL = 316, MM = 342, NHL = 513, and STS = 357, Total number of cases = 1528). Each case was supposed to have one matched control (match on ± 2 years and province of residence), however some problems were faced in recruiting controls in older age groups, specially 69 years and older, therefore we have 1506 controls. To analyze data for each specific tumour type, all controls from the entire case groups were used. For example in this report statistical analysis is based on 316 (68.4% of those contacted) HL cases and all 1506 (48.0% of those contacted) controls. The most common reasons for not participating were death, change of address and refusal for both study groups. The study had approximately five matched controls for each HL case.

This report is based solely on cases diagnosed with HL and to test the secondary hypothesis mentioned above. Incident cases among men, ages 19 years or over, with first diagnosis of HL (ICD-9 201) between September 1, 1991 to December 31, 1994 were eligible. Cases were ascertained from provincial Cancer Registries except in Quebec. For Quebec, hospital ascertainment was used. After physician consent was received, postal questionnaires and informed consent forms were mailed to potential cases. Control subjects were matched by age ± 2 years to be comparable with the age distribution of the entire case group [HL, NHL, MM, and STS] within each province of residence. Potential controls (men, 19 ages and older) selected at random within age constraints from the provincial Health Insurance records (Alberta, Saskatchewan, Manitoba, Quebec), computerized telephone listings (Ontario), or voters' lists (British Columbia). Postal questionnaires and informed consent forms were mailed to potential controls, and information collected from all of the participating control subjects was used in the statistical analyses of each cancer site. Deceased subjects were ineligible as either cases or controls.

The study design consisted of two stages: Stage 1 was a self-administered postal questionnaire; and Stage 2 was detailed pesticide exposure information collected via telephone interview. With permission, we modified a pesticide exposure questionnaire developed by Hoar et al. [33] to create these questionnaires.

The postal questionnaires captured demographic details, personal medical history, cigarette smoking history, lifetime occupational history and specific occupational exposures of interest. Each subject who reported 10 hours per year or more of exposure to pesticides (in any combination of compounds) as defined by the screening questions, and a 15% random sample of the remainder was mailed a list of pesticides (both chemical and brand names) and an information letter. Each subject was subsequently telephoned to obtain details of pesticide use. Exposure to individual herbicide, insecticides, fumigant, and fungicide was coded as 'Yes' if the response to following question 'Did you use it at work, in your home garden or as a hobby' was either work or home or hobby otherwise it was coded as 'No'. All individuals who had exposure less than 10 hours based on the postal questionnaire and were not a part of 15% random sample were considered as 'No exposure to individuals chemical listed in the telephone questionnaire.'

The telephone questionnaire characterized exposure to individual pesticides. The pesticide data were collected at several levels beginning with the broadest categories (*e.g*. minimal exposure, occupations with potential pesticide exposure) and progressing sequentially from major classes (*e.g*., herbicides); to chemical groups (*e.g*., phenoxy herbicides); and finally to individual components (*e.g*., 2,4-D, MCPA, and 2,4,5-T). The listed pesticides (Additional file 1: Table S1) were chosen for inclusion for the following reasons: (a) if the compound was ever registered for use in Canada and reviewed by the IARC; (b) if the pesticide was recently banned or restricted in Canada by the federal licensing agency; or (c) if the pesticide was commonly used in Canada for specific purposes [34-37].

### Ethical Approval

The letters of informed consent, the questionnaires and other written material provided to potential subjects were submitted to and approved by each of the relevant agencies in each province. For Saskatchewan, the University of Saskatchewan Biomedical Research Ethics Board (#89-12); for British Columbia, the University of British Columbia Behavioural Research Ethics Board (#B91-185); for Quebec, the University of McGill Human Ethics Board; for Alberta, the University of Alberta Health Research Ethics Board (Biomedical Panel); for Manitoba, the University of Manitoba Biomedical Research Ethics Board; and for Ontario, the University of Toronto Health Sciences Research Board approved the study protocol. All information that could be used to identify the individuals remained within the province of origin under the control of each province's principal investigator. After receiving permission to contact a potential case by the attending physician, a letter of informed consent and a questionnaire was mailed. Relatives of cases known to be deceased at ascertainment were not contacted because of the need to obtain detailed information concerning occupation and occupational exposures including a variety of types of pesticides. Control subjects were contacted directly by mail. Living control subjects were eligible.

### Definitions

Several definitions used in this manuscript are given below.

### Ethnicity

In the questionnaire, each individual was asked for birthplace (the country or part of the world) of each of his four grandparents. Since there have been reports that studies of pesticide exposure conducted among certain ethnic groups have produced higher odds ratios than similar types and doses of exposure among other ethnic groups, this question was included in our study. According to the information based on the ethnicity of the father's father, father's mother, mother's father, and mother's mother, the respondents' ethnicity was defined. Horne et al. [38], classified each individual predominantly in a particular ethnicity if he were 4/4 (all four grandparents) or 3/4 (three grandparents out of four) from that group. Any individual who had less than 3 grandparents from the same ethnic group was classified in the "other" category. According to that definition we formed seven ethnic categories. The composition of the categories is based on geography and some of the groups are quite broad due to aggregate grouping with small numbers. The ethnic categories are as follows: Scandinavian (Denmark, Finland, Norway and Sweden), Eastern European (Austria, Bulgaria, Czechoslovakia, Hungary, Poland, Romania, Serbia, Yugoslavia, Russia and Ukraine), Western European (Belgium, France, Greece, Germany, Holland, Italy, Luxembourg, Portugal, Spain and Switzerland), North American (Canada and United States), Asian (Asia, China, India, Japan, Korea, Laos, Pakistan, Philippines and Vietnam), British (Australia, England, Great Britain, Ireland, New Zealand, Scotland and Wales), and other (Adopted, Africa, American Indian, Central American, Europe, Iceland, Malta, Mexico, Mediterranean Sea, Middle East, Pacific Islands, Puerto Rico, South America, and West Indies).

### Pesticide

Pesticide is a generic term which refers to a host of compounds of diverse chemical structure and biological modes of action. In this study, the term pesticide refers primarily to herbicides, insecticides, fumigants and fungicides.

### Pesticide Exposure

We conducted a pilot study [39] in each provincial region to test questionnaires and to determine a working definition of pesticide exposure to distinguish between environmental (which includes bystander and incidental) and more intensive exposure. Non-occupational use of pesticides (home, garden, and hobby) was included. Based on the statistical analysis of pilot study data, it was decided that the most efficient definition of pesticide exposure, which discriminated (a) between incidental, bystander, and environmental exposure as compared with more intensive exposure and (b) between cases and controls, was a cumulative exposure = 10 hrs/year to any combination of pesticides. The screening questions in the postal questionnaire were used to prompt telephone interviews among those with cumulative exposure of = 10 hrs/year to any combination of herbicides, insecticides, fungicides, fumigants, and/or algicides.

### Medical History

Information on antecedent medical history, and family history of cancer was collected (see Table 1). Information was collected on infectious disease (bacterial and viral), allergies, acne, and immune disorders which includes autoimmune and inflammatory disorders, and asthma.

**Table 1:** Personal Medical History of HL Cases and Controls

	HL (n = 316)		Controls (n = 1506)		OR^a ^(95% Cl)
	n	%	n	%	

Asthma	22	7.0	107	7.1	0.90 (0.53, 1.54)
Acne	30	9.5	48	3.2	**2.05 (1.21, 3.48)**
Chicken pox	155	49.0	638	42.4	0.94 (0.71, 1.25)
Diabetes	12	3.8	99	6.6	1.08 (0.55, 2.09)
Hay fever	28	8.9	155	10.3	0.68 (0.43, 1.07)
Measles	153	48.4	888	59.0	**0.76 (0.57,0.99)**
Mumps	137	43.3	661	43.9	1.11 (0.85,1.47)
Mononucleuosis	23	7.3	48	3.2	1.30 (0.74, 2.72)
Rheumatoid arthritis	8	2.53	88	5.84	0.78 (0.36, 1.69)
Rheumatic fever	4	1.3	36	2.4	0.87 (0.29, 2.58)
Shingles	28	8.9	87	5.8	**2.16 (1.31,3.56)**
Ringworm	14	4.4	89	5.9	0.74 (0.40, 1.37)
Urinary tract infection	24	7.6	155	10.3	1.06 (0.66,1.72)
Whooping cough	30	9.5	220	14.6	0.90 (0.57, 1.40)
Allergies	77	24.4	378	25.1	0.81 (0.60, 1.11)
Allergy desensitization shots	19	6.6	114	8.1	0.66 (0.38, 1.14)
Patch skin test for allergy	14	5.0	60	4.3	1.14 (0.59, 2.20)
Treatment for head or body lice or scabies	21	7.3	124	8.7	0.68 (0.41, 1.15)
Tonsillectomy	76	26.5	429	30.1	1.02 (0.74, 1.41)
First degree relative with cancer	104	34.0	498	33.8	**1.79 (1.33, 2.42)**

OR^a ^calculated with strata for the variables of age (5 year groups) and province of residence.

Statistically significant results are bold.

### Family history of cancer

Family is restricted to first degree relatives (parents, siblings, and offspring) who share, on average, one half of the respondents' genes. Family excludes half-siblings, adopted siblings and children, step-children and all other non-related individuals. Family excludes the index subjects.

### Statistical Analyses

Data were entered into a custom designed SPSS-data entry program. Results were presented as frequencies for categorical variables and mean, standard deviation (SD) or standard error (SE) for continuous variables for cases and controls separately. A bivariate analysis was conducted to determine the association between each explanatory variable and HL outcome. Based on this model building procedure explanatory variables with p < 0.20 were selected for the multivariate model. Statistically significant (p < 0.05) variables and important explanatory variables were considered for the final multivariate model adjusting for age and province of residence. Frequency matching by five-year intervals and geographical region was used in order to conduct conditional logistic regression (using strata defined on 'cross-classifying 5-years intervals and province of residence') to compute adjusted odds ratios (OR_adj_) and 95% confidence intervals (95% CI).

## Results

The cases were significantly younger (mean age ± standard deviation (SD): 40.2 ± 15.9) compared to controls (54.1 ± 16.4). 44.6% HL cases were younger than 35 years; 31.6% cases were in the age group = 35 and <50, and 23.4% were 50 years and older. There were no significant difference between HL cases and controls by region (Additional file 2: Table S2). The distribution of ethnic groups varied significantly among different regions of Canada (Additional file 3: Table S3). Based on the population case-control study conducted during 1991–1994, there were 15% of Eastern European descent in the Prairies (Alberta, Saskatchewan, and Manitoba); 12% Western European descent in Ontario; 83% North American descent in Quebec; 3% Asian descent in Ontario and British Columbia and 30% British descent in British Columbia. Overall, 28% of cases and 27% of controls were categorized into "Other" category. The distribution of ethnicities within the "Other" category is reported in a separate table, which is presented in the Additional file 4: Table S4.

Personal medical history of cases and population-based controls are summarized and compared in Table 1. Most of the personal medical history variables were not found to be associated with risk of HL. Those diagnosed with acne (OR_adj _= 2.05, 95% CI = 1.21 – 3.48) and with shingles (OR_adj _= 2.16, 95% CI = 1.31 – 3.56) were positively associated with risk of HL. Those diagnosed with measles were negatively associated with risk of HL (OR_adj _= 0.76, 95% CI = 0.57 – 0.99). A statistically significant (OR_adj _= 1.79, 95% CI = 1.33 – 2.42) increased risk for HL was found to be associated with a history of first-degree relatives with cancer. In addition to that herbicide Dichlorprop (OR_adj _= 3.85, 95% CI = 1.19 – 12.47) was positively associated with increasing the risk of HL (Table 2). Based on conditional logistic multivariable regression modeling, the following factors independently increased the risk of HL: Eastern European descent, exposure to Dichlorprop, immediate family history of cancer, personal medical history of acne and shingles. Those diagnosed with measles or treated with allergy desensitization shots were negatively associated with HL (Table 3). The risk of HL was significantly greater among the Eastern European (OR_adj _= 1.82, 95% CI = 1.02 – 3.25) and Western European (OR_adj _= 1.62, 95% CI = 0.95 – 2.76) descent populations in Canada.

**Table 2:** Results based on univariate conditional logistic regression models.

Variable	Sample size	OR^a ^(95% CI)
**Ethnicity **(reference is North American)		
Scandinavian	1822 (1506 Controls; 316 Cases)	0.34 (0.04, 2.80)
Eastern European	1822 (1506 Controls; 316 Cases)	1.42 (0.83, 2.44)
Western European	1822 (1506 Controls; 316 Cases)	1.42 (0.90, 2.40)
Asian	1822 (1506 Controls; 316 Cases)	0.14 (0.02, 1.07)
British	1822 (1506 Controls; 316 Cases)	1.04 (0.66, 1.65)
Other	1822 (1506 Controls; 316 Cases)	0.94 (0.67, 1.33)
**Herbicides**		
Dichlorprop	1822 (1506 Controls; 316 Cases)	3.85 (1.19–12.47)
**Personal Medical History**		
Diagnosed with measles	1822 (1506 Controls; 316 Cases)	**0.76 (0.57, 1.00)**
Ever diagnosed with acne	1822 (1506 Controls; 316 Cases)	**2.05 (1.21, 3.48)**
Diagnosed with shingles	1822 (1506 Controls; 316 Cases)	**2.16 (1.31, 3.56)**
First degree relative with cancer	1776 (1471 Controls; 306 Cases)	**1.79 (1.33,2.42)**
Allergy desensitization shots	1705 (1417 Controls; 288 Cases)	0.66 (0.38, 1.14)

Results reported for variables with p < 0.20

OR^a ^calculated with strata for the variables of age (5 year groups) and province of residence.

Statistically significant (p < 0.05) results are bold.

**Table 3:** Results based on multivariable conditional logistic regression

Variable	OR^a ^(95% CI)
**Ethnicity **(reference is North American)	
Scandinavian	0.46 (0.05,3.71)
Eastern European	**1.82 (1.02,3.25)**
Western European	1.62 (0.95,2.76)
Asian	0.17 (0.02,1.33)
British	1.00 (0.60,1.67)
Other	1.03 (0.70,1.50)
**Herbicides**	
Dichlorprop	**6.35 (1.56,25.92)**
**Personal Medical History**	
Diagnosed with measles	**0.72 (0.53,0.98)**
Ever diagnosed with acne	**2.12 (1.19,3.78)**
Diagnosed with shingles	**2.41 (1.38,4.22)**
First degree relative with cancer	**1.93 (1.40,2.65)**
Allergy desensitization shots	**0.55 (0.30,0.99)**

OR^a ^calculated with strata for the variables of age (5 year groups) and province of residence.

Statistically significant (p < 0.05) results are bold.

## Discussion

The concept of ethnicity, ancestry, and race are widely used in molecular epidemiologic research, and are often based on the assumption that these correlate with increased genetic homogeneity among people claiming a similar identity. The important finding of this report is that compared to North Americans: (i) Eastern Europeans (p = 0.05) and Western European (p = 0.09) were significantly at higher risk of developing HL, and that (ii) Asians were significantly (p = 0.09) at lower risk of developing HL. Findings from descriptive and multivariate logistic regression analyses of our study suggest incidence of HL was significantly higher [OR, 95% C.I.: 1.82 (1.02, 3.25)] compared to subject of North Americans descent. Incidence of HL was significantly (borderline) higher in Western European compared to North Americans; (ii) exposure to herbicide dichlorprop is significantly associated with the increased risk of HL; (iii) personal medical history variables: diagnosed with measles and ever had allergy desensitization shots were significantly associated with decreased incidence of HL. In contrast, some of the other personal medical history variables: 'Ever diagnosed with acne', 'Diagnosed with shingles', and 'First degree relatives diagnosed with cancer' were significantly associated with increased incidence of HL. Our results support findings of high incidence of lymphomas and other cancers in European descent populations. For example, Leukemia rates are higher in Americans of European descent than among those of any other race/ethnicity [40], and as reported by Spallek et al [41] that for acute non-lymphocytic leukemia, Hodgkin's disease 1.34 (1.13–1.59) and Non-Hodgkin/Burkitt lymphoma, the proportions are slightly increased for Turkish children (A study based at the German Childhood Cancer Registry). Our findings also suggest that that there is some genetic resistance to HL in Asians but also show that environmental factors have some influence, which are consistent with the findings reported by Zahm et al [24]. Genetic resistance to HL in Asian population is also supported by another study, which uniformly collected population-based data from the US and Asia, and authors reported that given environmental and lifestyle differences between the US and Asia, the consistently low rates of HL in Asians suggest genetic resistance to disease development [11].

As reported by other authors, the incidence of HL varies in different ethnic groups [10]; and incidence rates increased with advancing age at diagnosis and that white adolescents 15–19 years old exhibit the highest rates [42]. In another study Cozen et al. reported that blacks experience a slightly higher incidence of HL than whites in childhood but a lower incidence in young adulthood [43]. Ethnicity can serve as surrogate measures to identify high-risk groups. Groups that have a particularly high incidence or strong familial aggregation of disease may represent an optimal resource in which to identify or characterize disease genes. Another article based on Canadian Case-control population study showed that there is a familial aggregation among HL cases (manuscript accepted for publication in BMC Cancer).

It has been reported that HL may be a rare consequence of a common infection, with the probability of oncogenesis increasing with age at the time of infection. Patients with a history of infectious mononucleosis, a disease associated with late exposure to EBV, have a threefold increase in the risk of HL [44]. We did not collect information on EBV; however, a descriptive cross tabs analysis showed a significant association between diagnosis of mononucleosis and incidence of HL (7.3% HL cases were diagnosed with mononucleosis compared to 3.2% controls diagnosed with mononucleosis). No significant (p = 0.36) association was observed when a conditional logistic regression stratified by province and age was conducted, and diagnosis of mononucleosis did not enter into the multivariable conditional logistic regression. As reported by Hardell and Bengtsson [45], one aspect in the aetiology of HL is a possible interaction between chemical exposure, and viral infections such as EBV. Because of lack of information on EBV, we investigated the interaction between mononucleosis and pesticide exposure, which was not significant. Diagnosis of allergies were inversely associated with HL at p < 0.2 (Table 2), which is consistent with findings of earlier studies [20-23]. Our data showed that previous history of desensitization (those who were diagnosed with allergies, 27.2% has desensitization shots) was negatively associate with risk of HL. Diagnosis of shingles was positively associated with increased risk of HL, which could be due to weak immune system as a consequence of diagnosis of shingles. Most of the studies in the literature have reported that diagnosis of shingles is usually a consequence of HL, however in our case-control study information on previous medical conditions (including diagnosis of shingles) was collected.

Not only heredity and genetic factors but also environmental or non-genetic factors may increase the risk of Hodgkin lymphoma. Association between exposure to pesticides and increased risk of HL is controversial. Some studies have reported no association between exposure to (i) phenoxy acid herbicides and increased risk of HL [24,26,31], (ii) insecticides and increased risk of HL [31], while other studies have reported an association between exposure to phenoxy herbicides and HL [27,28]. Our study showed a positive significant association between exposure to Dichlorprop and HL, when ethnicity and some of the selected medical history variables were already in the model.

Like any other epidemiological case-control study, our study had strengths and limitations. Some of the strengths of our study are the following: it included a large number of cases and controls with approximately 5 controls per case; we conducted a pilot study to determine a suitable definition of pesticide exposure and we tested the study procedures prior to implementation; a reference pathologist reviewed the slides and confirmed that the diagnosis of 316 HL cases and slides were unavailable for the remainder; pesticide exposure was defined by 10 hours or more exposure per year to delineate between environmental and more intense exposure to pesticides, and, finally, the study includes men who are exposed to pesticides both indoors such as in animal confinement buildings and grain elevators and manufacturing and also outdoors such as in grain farming and commercial applications.

The main limitation of our study was the lack of information on Epstein-Barr virus (EBV). HL has been suspected to have an infectious precursor. Recent molecular studies have identified EBV latent infection in up to 50% of HL tumours [46]. One other limitation of our study was the lack of information on income, so we were unable to explore the relationship between HL and socioeconomic status.

## Conclusion

In Canada, compared to North Americans descendents, the risk of HL was significantly greater among the Eastern European and Western European descent population. Our results related to association between ethnicity and HL support the findings reported by other researchers. Our data showed that subjects who were diagnosed with measles or had allergy desensitization shots negatively associated with the incidence of HL; and other medical conditions, ever diagnosed with acne, and positive family history of cancer were positively associated with the incidence of HL.

## Abbreviations

(NHL): Non-Hodgkin's Lymphoma; (ICD): International Classification of Diseases; (STS): Soft Tissue Sarcoma; (HL): Hodgkin lymphoma; (MM): Multiple Myeloma; (EBV): Epstein-Barr Virus; (SD): Standard deviation; (SE): Standard Error; (OR_adj_): Adjusted Odds Ratio; (95% CI): 95% confidence Interval; (IARC): International Agency for Research on Cancer.

## Competing interests

The authors declare that they have no competing interests.

## Authors' contributions

PP was involved at the grant writing stage, participated in the design of the study, and questionnaires development. PP was the national coordinator and biostatistician for this study. PP trained data managers from the six provinces about data entry process, and supervised every stage of data cleaning. PP made significant contributions in the original statistical analysis required for the manuscript and preparation of the manuscript. CK made significant contributions in continuing with the statistical analysis and preparation of the manuscript. JS was the co-investigator who represented British Columbia and participated in the design of the study and supervised data collection for the province of British Columbia. JD and HM were co-principal investigators of the study. JD's main contribution was at the grant writing stage and questionnaires development. HM's main contribution was at the grant writing stage, design of the study, questionnaires development, coordination of the study. HM's developed the concept of including ethnicity, and medical history variables in the questionnaire in addition to the risk factors of primary interest. All authors read and approved the final manuscript.

## Pre-publication history

The pre-publication history for this paper can be accessed here:

http://www.biomedcentral.com/1471-2407/9/141/prepub

## Supplementary Material

Additional file 1 **Table S1: Major Chemical Classes**. List of pesticides (herbicides, insecticides, fungicides, fumigants) included in the study. Click here for file

Additional file 2 **Table S2: HL cases and controls and socio-demographics and other characteristics stratified by region**. Distribution of socio-demographics, pesticide exposure and smoking history of HL cases and controls. Click here for file

Additional file 3 **Table S3: Number of HL cases and controls stratified by ethnicity and region**. Distribution of ethnicity stratified by region for HL cases and controls. Click here for file

Additional file 4 **Table S4: Number of HL cases and controls stratified by grandparent's ethnic groups**. Country or ethnicity of grandparents (mother's mother, mother's father, father's mother, father's father) of HL cases and controls. Click here for file
