# Supplementary material for: Ethnicity and incidence of Hodgkin lymphoma in Canadian population
Source: BMC Cancer. 2009 May 11;9:141. doi: 10.1186/1471-2407-9-141 (PMC2690601; doi:10.1186/1471-2407-9-141)
Supplement: Additional file 1 — Table S1: Major Chemical Classes. List of pesticides (herbicides, insecticides, fungicides, fumigants) included in the study. [file 1471-2407-9-141-S1.doc]

**Table S1. Major Chemical Classes**

| For Herbicides | For Insecticides | For Fungicides | For Fumigants |
| --- | --- | --- | --- |
| Phenoxyherbicides | Carbamates | Amide | Malathion |
| Individual phenoxyherbicides | Individual carbamate insecticides | Individual amide fungicides | Carbon tetrachloride |
| 2,4-D | Carbaryl | Captan |  |
| Mecoprop | Carbofuran | Vitavax |  |
| MCPA | Methomyl |  |  |
| Diclofopmethyl |  | Aldehyde |  |
|  | Organochlorine | Individual aldehyde fungicides |  |
| Phosphonic acid | Individual organochlorine (1) insecticides | Formaldehyde |  |
| Individual phosphonic herbicides | Chlordane |  |  |
| Glyphosate (Round-up) | Lindane | Mercury Containing |  |
|  | Aldrin | Mercury-containing fungicides |  |
| Thiocarbamates |  | Mercury dust |  |
| Individual thiocarbamate  herbicides | Organochlorine  diphenylchlorides,*^e^* | Mercury liquid |  |
| Diallate | Individual organochlorine diphenylchlorides |  |  |
|  | Methoxychlor | Sulphur Compounds |  |
| Phenols: Bromoxynil,*^f^* exposed | DDT |  |  |
|  |  |  |  |
| Dicamba | Organophosphorus,*^f^* exposed |  |  |
| Individual dicamba herbicites | Individual organophosphorus insecticides |  |  |
| Dicamba (Banvel or Target) | Malathion |  |  |
|  | Dimethoate |  |  |
| Dinitroaniline | Diazinon |  |  |
| Individual dinitroanilinc herbicides |  |  |  |
| Trifluralin |  |  |  |
