# Supplementary material for: Ethnicity and incidence of Hodgkin lymphoma in Canadian population
Source: BMC Cancer. 2009 May 11;9:141. doi: 10.1186/1471-2407-9-141 (PMC2690601; doi:10.1186/1471-2407-9-141)
Supplement: Additional file 2 — Table S2: HL cases and controls and socio-demographics and other characteristics stratified by region. Distribution of socio-demographics, pesticide exposure and smoking history of HL cases and controls. [file 1471-2407-9-141-S2.doc]

**Table S2:**  HL Cases and Controls and socio-demographics and other characteristics stratified by region.

| Variable | Ontario (n=707) | | Quebec (n=343) | | Prairies (n=491) | | British Columbia (n=281) | |  | | Total |
| --- | --- | --- | --- | --- | --- | --- | --- | --- | --- | --- | --- |
|  | Cases (n=122)  n(%) | Controls  (n=585) n(%) | Cases  (n=52) n(%) | Controls  (n=291) n(%) | Cases  (n=91) n(%) | Controls  (n=400) n(%) | Cases  (n=51)  n(%) | Controls  (n=230) n(%) | Cases  (n=316)  n(%) | Controls  (n=1506) n(%) |  |
| **Age, yr** |  |  |  |  |  |  |  |  |  |  |  |
| <30 | 37 (33.0) | 51 (9.4) | 16 (33.3) | 29 (11.0) | 32 (37.2) | 30 (8.0) | 8 (16.3) | 11 (5.1) | 93 (31.5) | 121 (8.7) | 214 |
| 30-39 | 26 (23.2) | 105 (19.3) | 10 (20.8) | 32 (12.2) | 27 (31.4) | 37 (9.9) | 12 (24.5) | 10 (4.6) | 75 (25.4) | 184 (13.2) | 259 |
| 40-49 | 19 (16.7) | 95 (17.5) | 10 (20.8) | 58 (22.0) | 12 (13.9) | 43 (11.5) | 15 (30.6) | 25 (11.5) | 56 (20.0) | 221 (15.8) | 277 |
| 50-59 | 9 (8.0) | 80 (14.7) | 5 (10.4) | 38 (14.4) | 4 (4.6) | 62 (16.6) | 7 (14.3) | 34 (15.7) | 25 (8.5) | 214 (15.3) | 239 |
| >60 | 21 (18.7) | 213 (39.1) | 7 (14.6) | 106 (40.3) | 11 (12.8) | 201 (53.9) | 7 (14.3) | 137 (63.1) | 46 (15.6) | 657 (47.0) | 703 |
| Mean ±SD | 41±17 | 51±16 | 39±16 | 51±16 | 38±16 | 57±16 | 43±14 | 60±15 | 54 ± 16 | 40 ± 16 | - |
| **Residence on a farm at any time** |  |  |  |  |  |  |  |  |  |  |  |
| Yes | 33 (27.1) | 215 (36.8) | 11 (21.1) | 96 (32.9) | 52 (57.1) | 270 (67.5) | 21 (41.2) | 92 (40.0) | 117 (37.0) | 673 (44.7) | 790 |
| No (reference) | 89 (72.9) | 370 (63.2) | 41 (78.9) | 195 (67.1) | 39 (42.9) | 130 (32.5) | 30 (58.8) | 138 (60.0) | 199 (63.0) | 833 (55.3) | 1032 |
|  |  |  |  |  |  |  |  |  |  |  |  |
| **Pesticide exposure**  (screening question) |  |  |  |  |  |  |  |  |  |  |  |
| <10 hr/yr (reference) | 99 (81.1) | 452 (77.3) | 43 (82.7) | 239 (82.1) | 59 (64.8) | 259 (64.8) | 39 (76.5) | 192 (83.5) | 240 (76.0) | 1142 (75.8) | 1382 |
| ≥ 10 hr/yr | 23 (18.9) | 133 (22.7) | 9 (17.3) | 52 (17.9) | 32 (35.2) | 141 (35.2) | 12 (23.5) | 38 (16.5) | 76 (24.0) | 364 (24.2) | 440 |
| **Smoking History** (missing=11) |  |  |  |  |  |  |  |  |  |  |  |
| Nonsmoker (reference) | 44 (36.4) | 226 (38.9) | 21 (40.4) | 94 (32.6) | 46 (50.5) | 135 (34.0) | 18 (35.3) | 72 (31.3) | 129 (40.9) | 527 (35.2) | 656 |
| Ex-smoker | 50 (41.3) | 230 (39.6) | 11 (21.1) | 123 (42.7) | 23 (25.3) | 179 (45.1) | 18 (35.3) | 115 (50.0) | 102 (32.4) | 647 (43.3) | 749 |
| Current smoker | 27 (22.3) | 125 (21.5) | 20 (38.5) | 71 (24.6) | 22 (24.2) | 83 (20.9) | 15 (29.4) | 43 (18.7) | 84 (26.7) | 322 921.5) | 406 |
| **Education level** |  |  |  |  |  |  |  |  |  |  |  |
| University and Vocational | 43 (35.8) | 232 (40.2) | 29 (55.8) | 163 (56.6) | 40 (44.0) | 224 (56.8) | 17 (33.3) | 104 (45.6) | 129 (41.1) | 723 (48.6) | 852 |
| University | 45 (37.5) | 157 (27.2) | 8 (15.4) | 37 (12.8) | 20 (21.9) | 65 (16.5) | 11 (21.6) | 51 (22.4) | 84 (26.8) | 310 (20.8) | 394 |
| Vocational | 25 (20.8) | 154 (26.7) | 13 (25.0) | 61 (21.2) | 26 (28.6) | 87 (22.1) | 17 (33.3) | 56 (24.6) | 81 (25.8) | 358 (24.1) | 439 |
| Elementary/High school (reference) | 7 (5.8) | 34 (5.9) | 2 (3.8) | 27 (9.4) | 5 (5.5) | 18 (4.6) | 6 (11.8) | 17 (7.5) | 20 (6.4) | 96 (6.5) | 116 |
