# Supplementary material for: Ethnicity and incidence of Hodgkin lymphoma in Canadian population
Source: BMC Cancer. 2009 May 11;9:141. doi: 10.1186/1471-2407-9-141 (PMC2690601; doi:10.1186/1471-2407-9-141)
Supplement: Additional file 3 — Table S3: Number of HL cases and controls stratified by ethnicity and region. Distribution of ethnicity stratified by region for HL cases and controls. [file 1471-2407-9-141-S3.doc]

**Table S3:** Number of HL cases and controls stratified by ethnicity and region

| Ethnicity | Ontario  (n=707) | | Quebec  (n=343) | | Prairies  (n=491) | | British Colombia  (n=281) | | Total  (n=1822) | |
| --- | --- | --- | --- | --- | --- | --- | --- | --- | --- | --- |
|  | Cases (n=122)  n(%) | Controls  (n=585) n(%) | Cases  (n=52) n(%) | Controls  (n=291) n(%) | Cases  (n=91) n(%) | Controls  (n=400) n(%) | Cases  (n=51)  n(%) | Controls  (n=230) n(%) | Cases  (n=316)  n(%) | Controls  (n=1506)  n(%) |
| Scandinavian | 0 (0.0) | 6 (1.0) | 0 (0.0) | 0 (0.0) | 1 (1.1) | 9 (2.2) | 0 (0.0) | 9 (3.9) | 1 (0.3) | 24 (1.6) |
| Eastern European | 12 (9.8) | 32 (5.5) | 2 (3.8) | 8 (2.7) | 10 (10.9) | 63 (15.7) | 1 (1.9) | 21 (9.1) | 25 (7.9) | 124 (8.2) |
| Western European | 16 (13.1) | 72 (12.3) | 3 (5.8) | 8 (2.7) | 10 (10.9) | 23 (5.7) | 2 (3.9) | 19 (8.3) | 31 (9.8) | 122 (8.1) |
| North American | 44 (36.1) | 199 (34.0) | 42 (80.8) | 244 (83.8) | 33 (36.3) | 87 (21.7) | 13 (25.5) | 39 (16.9) | 132 (41.8) | 569 (37.8) |
| Asian | 0 (0.0) | 18 (3.1) | 0 (0.0) | 1 (0.3) | 0 (0.0) | 3 (0.7) | 1 (1.9) | 8 (3.5) | 1 (0.3) | 30 (1.9) |
| British | 17 (13.9) | 96 (16.4) | 1 (1.9) | 5 (1.7) | 5 (5.5) | 63 (15.7) | 14 (27.4) | 71 (30.9) | 37 (11.7) | 235 (15.6) |
| Other | 33 (27.0) | 162 (27.7) | 4 (7.7) | 25 (8.6) | 32 (35.2) | 152 (38.0) | 20 (39.2) | 63 (27.4) | 89 (28.2) | 402 (26.7) |
