# Supplementary material for: Ethnicity and incidence of Hodgkin lymphoma in Canadian population
Source: BMC Cancer. 2009 May 11;9:141. doi: 10.1186/1471-2407-9-141 (PMC2690601; doi:10.1186/1471-2407-9-141)
Supplement: Additional file 4 — Table S4: Number of HL cases and controls stratified by grandparent's ethnic groups. Country or ethnicity of grandparents (mother's mother, mother's father, father's mother, father's father) of HL cases and controls. [file 1471-2407-9-141-S4.doc]

**Table S4:** Number of HL cases and controls stratified by grandparent’s ethnic groups

| Country/Ethnicity | Cases  (n=316) | | | | Controls  (n=1506) | | | |
| --- | --- | --- | --- | --- | --- | --- | --- | --- |
|  | Mother’s Mother born  n (%) | Mother’s father born  n (%) | Father’s mother born  n (%) | Father’s father born  n (%) | Mother’s Mother born  n (%) | Mother’s father born  n (%) | Father’s mother born  n (%) | Father’s father born  n (%) |
| **Africa^#^** | 1 (0.3) | 1 (0.3) | 1 (0.3) | 1 (0.3) | 5 (0.3) | 7 (0.5) | 6 (0.4) | 5 (0.3) |
| **American Indian^#^** | 0 (0.0) | 0 (0.0) | 0 (0.0) | 0 (0.0) | 1 (0.1) | 0 (0.0) | 0 (0.0) | 0 (0.0) |
| Australia | 0 (0.0) | 0 (0.0) | 0 (0.0) | 0 (0.0) | 0 (0.0) | 0 (0.0) | 0 (0.0) | 1 (0.1) |
| Austria | 1 (0.3) | 0 (0.0) | 3 (0.9) | 3 (0.9) | 9 (0.6) | 7 (0.5) | 13 (0.9) | 14 (0.9) |
| Belgium | 2 (0.6) | 1 (0.3) | 2 (0.6) | 1 (0.3) | 5 (0.3) | 3 (0.2) | 6 (0.4) | 6 (0.4) |
| Canada | 147 (46.5) | 137 (43.4) | 144 (45.6) | 124 (39.2) | 641 (42.6) | 613 (40.7) | 627 (41.6) | 615 (40.8) |
| **Central America^#^** | 0 (0.0) | 0 (0.0) | 0 (0.0) | 0 (0.0) | 3 (0.2) | 2 (0.1) | 3 (0.2) | 2 (0.1) |
| China | 2 (0.6) | 2 (0.6) | 1 (0.3) | 1 (0.3) | 14 (0.6) | 17 (1.1) | 17 (1.1) | 15 (1.0) |
| Czechoslovakia | 2 (0.6) | 2 (0.6) | 1 (0.3) | 1 (0.3) | 4 (0.3) | 7 (0.5) | 10 (0.7) | 8 (0.5) |
| Denmark | 0 (0.0) | 2 (0.6) | 2 (0.6) | 3 (0.9) | 3 (0.2) | 4 (0.3) | 8 (0.5) | 8 (0.5) |
| England | 19 (6.0) | 26 (8.2) | 32 (10.1) | 31 (9.8) | 183 (12.2) | 190 (12.6) | 192 (12.8) | 194 (12.9) |
| Finland | 0 (0.0) | 0 (0.0) | 1 (0.3) | 0 (0.0) | 10 (0.7) | 9 (0.6) | 5 (0.3) | 6 (0.4) |
| France | 1 (0.3) | 2 (0.6) | 1 (0.3) | 2 (0.6) | 12 (0.8) | 15 (1.0) | 12 (0.8) | 16 (1.1) |
| Germany | 9 (2.9) | 12 (3.8) | 10 (3.2) | 11 (3.5) | 54 (3.6) | 58 (3.8) | 48 (3.2) | 48 (3.2) |
| Great Britain | 1 (0.3) | 1 (0.3) | 1 (0.3) | 1 (0.3) | 17 (1.1) | 18 (1.2) | 13 (0.9) | 13 (0.9) |
| Greece | 1 (0.3) | 2 (0.6) | 1 (0.3) | 2 (0.6) | 2 (0.1) | 2 (0.1) | 3 (0.2) | 3 (0.2) |
| Holland/ Netherlands | 4 (1.3) | 4 (1.3) | 28 (1.9) | 5 (1.6) | 28 (1.9) | 27 (1.8) | 4 (1.3) | 30 (2.0) |
| Hungary | 3 (0.9) | 3 (0.9) | 0 (0.0) | 0 (0.0) | 9 (0.6) | 9 (0.6) | 9 (0.6) | 9 (0.6) |
| **Iceland^#^** | 0 (0.0) | 0 (0.0) | 0 (0.0) | 1 (0.3) | 4 (0.3) | 2 (0.1) | 1 (0.1) | 1 (0.1) |
| India | 0 (0.0) | 0 (0.0) | 0 (0.0) | 1 (0.3) | 12 (0.8) | 12 (0.8) | 10 (0.7) | 11 (0.7) |
| Ireland | 8 (2.5) | 11 (3.5) | 2 (0.6) | 9 (2.8) | 40 (2.7) | 46 (3.1) | 42 (2.8) | 53 (3.5) |
| Italy/Sicely/Sardinia | 18 (5.7) | 17 (5.4) | 19 (6.0) | 19 (6.0) | 40 (2.7) | 41 (2.7) | 37 (2.5) | 39 (2.6) |
| Japan | 0 (0.0) | 0 (0.0) | 0 (0.0) | 0 (0.0) | 4 (0.3) | 4 (0.3) | 5 (0.3) | 5 (0.3) |
| **Mexico^#^** | 0 (0.0) | 0 (0.0) | 0 (0.0) | 0 (0.0) | 0 (0.0) | 0 (0.0) | 0 (0.0) | 1 (0.1) |
| **Middle East^#^** | 1 (0.3) | 2 (0.6) | 1 (0.3) | 1 (0.3) | 7 (0.5) | 7 (0.5) | 9 (0.6) | 9 (0.6) |
| Norway | 2 (0.6) | 1 (0.3) | 1 (0.3) | 2 (0.6) | 9 (0.6) | 10 (0.7) | 12 (0.8) | 9 (0.6) |
| **Pacific Islands^#^** | 0 (0.0) | 0 (0.0) | 0 (0.0) | 0 (0.0) | 1 (0.07) | 1 (0.1) | 1 (0.1) | 1 (0.1) |
| Poland | 7 (2.2) | 9 (2.8) | 8 (2.5) | 6 (1.9) | 33 (2.2) | 32 (2.1) | 36 (2.4) | 35 (2.3) |
| Portugal | 1 (0.3) | 1 (0.3) | 1 (0.3) | 1 (0.3) | 10 (0.7) | 10 (0.7) | 10 (0.7) | 10 (0.7) |
| Romania | 2 (0.6) | 2 (0.6) | 3 (0.9) | 3 (0.9) | 4 (0.3) | 4 (0.3) | 6 (0.4) | 6 (0.4) |
| Russia/ USSR | 6 (1.9) | 6 (1.9) | 7 (2.2) | 7 (2.2) | 36 (2.4) | 36 (2.4) | 32 (2.1) | 35 (2.3) |
| Serbia | 0 (0.0) | 0 (0.0) | 0 (0.0) | 0 (0.0) | 3 (0.2) | 3 (0.2) | 2 (0.1) | 2 (0.1) |
| Scotland | 16 (5.1) | 12 (3.8) | 16 (4.6) | 18 (5.7) | 68 (4.5) | 78 (5.1) | 70 (4.7) | 68 (4.5) |
| **South America^#^** | 1 (0.3) | 2 (0.6) | 0 (0.0) | 0 (0.0) | 2 (0.1) | 2 (0.1) | 3 (0.20 | 3 (0.2) |
| Spain | 0 (0.0) | 0 (0.0) | 0 (0.0) | 0 (0.0) | 2 (0.1) | 1 (0.1) | 1 (0.1) | 1 (0.1) |
| Sweden | 3 (0.9) | 1 (0.3) | 1 (0.3) | 0 (0.0) | 14 (0.9) | 14 (0.9) | 14 (0.9) | 15 (1.0) |
| Switzerland | 0 (0.0) | 0 (0.0) | 1 (0.3) | 1 (0.3) | 3 (0.2) | 2 (0.1) | 2 (0.1) | 3 (0.2) |
| Ukraine | 9 (2.9) | 10 (3.2) | 11 (3.5) | 12 (3.8) | 50 (3.3) | 52 (3.4) | 44 (2.9) | 48 (3.2) |
| United states | 14 (4.4) | 13 (4.1) | 10 (3.2) | 16 (5.1) | 41 (2.7) | 39 (2.6) | 52 (3.4) | 51 (3.4) |
| Wales | 3 (0.9) | 3 (0.9) | 2 (0.6) | 3 (0.9) | 12 (0.8) | 16 (1.1) | 9 (0.6) | 10 (0.7) |
| **West Indies^#^** | 1 (0.3) | 1 (0.3) | 1 (0.3) | 1 (0.3) | 5 (0.3) | 4 (0.3) | 4 (0.3) | 4 (0.3) |
| Yugoslavia | 3 (0.9) | 3 (0.9) | 2 (0.6) | 2 (0.6) | 5 (0.3) | 5 (0.3) | 6 (0.4) | 7 (0.5) |
| **Adopted^#^** | 1 (0.3) | 1 (0.3) | 1 (0.3) | 1 (0.3) | 1 (0.1) | 1 (0.1) | 2 (0.1) | 2 (0.1) |
| **Unknown^#^** | 28 (8.9) | 26 (8.2) | 25 (7.9) | 26 (8.2) | 100 (6.6) | 98 (6.5) | 96 (6.4) | 84 (5.6) |

^#^ “Other” category includes Africa, American Indian, Central America, Iceland, Mexico, Middle East, Pacific Islands, South American, West Indies, Adopted and unknown ethnicity for HL cases and controls. The ethnic categories according to our definition are as follows: Scandinavian (Denmark, Finland, Norway and Sweden), Eastern European (Austria, Bulgaria, Czechoslovakia, Hungary, Poland, Romania, Serbia, Yugoslavia, Russia and Ukraine), Western European (Belgium, France, Greece, Germany, Holland, Italy, Luxembourg, Portugal, Spain and Switzerland), North American (Canada and United States), Asian (Asia, China, India, Japan, Korea, Laos, Pakistan, Philippines and Vietnam), British ( Australia, England, Great Britain, Ireland, New Zealand, Scotland and Wales) if the person was 4/4 (all four grandparents) or 3/4 (three grandparents out of four) from these groups.
